# Morphological and Transcriptional Characteristics of the Symbiotic Interaction between *Pinus massoniana* and *Suillus bovinus*

**DOI:** 10.3390/jof8111162

**Published:** 2022-11-03

**Authors:** Wanyan Feng, Xueguang Sun, Guijie Ding

**Affiliations:** 1 Institute for Forest Resources & Environment of Guizhou, Guizhou University, Guiyang 550025, China; 2 Key Laboratory of Forest Cultivation in Plateau Mountain of Guizhou Province, Guizhou University, Guiyang 550025, China; 3 College of Forestry, Guizhou University, Guiyang 550025, China

**Keywords:** ectomycorrhiza, morphogenesis, transcriptome, phenylpropanoid, Hartig net

## Abstract

Ectomycorrhiza (ECM) function has been well studied; however, there is little detailed information regarding the establishment of ECM symbioses. We investigated the morphological and transcriptional changes that occur during the establishment of the *Pinus massoniana*–*Suillus bovinus* ECM. *S. bovinus* promoted the growth of *P. massoniana* via the release of volatile organic compounds and exudates during the pre-symbiotic stage. Exudate-induced effects showed host plant specificity. At seven days post-inoculation (dpi), the mycelium started to penetrate *P. massoniana* roots. At 28 dpi, the Hartig net and mantle formed. At the pre-symbiotic stage, most differentially expressed genes in *P. massoniana* roots were mapped to the biosynthesis of secondary metabolites, signal transduction, and carbohydrate metabolism. At the symbiotic stage, *S. bovinus* colonization induced the reprogramming of pathways involved in genetic information processing in *P. massoniana*, particularly at the Hartig net and mantle formation stage. Phenylpropanoid biosynthesis was present at all stages and was regulated via *S. bovinus* colonization. Enzyme inhibitor tests suggested that hydroxycinnamoyl-CoA shikimate/quinate transferase is involved in the development of the Hartig net. Our findings outline the mechanism involved in the *P. massoniana*–*S. bovinus* ECM. Further studies are needed to clarify the role of phenylpropanoid biosynthesis in ECM formation.

## 1. Introduction

An ectomycorrhiza (ECM) is a mutualistic association formed by ECM fungi and tree roots [1,2]. In this symbiotic relationship, ECM fungi improve host growth and fitness by promoting nutrient absorption and enhancing resistance to biotic stresses (such as pests and diseases) and abiotic stresses (such as drought and heavy metals) [3,4]. In exchange, fungi rely on the carbohydrates provided by their plant partners for vegetative growth and fruit body differentiation [5,6,7]. In addition, many ECM fungi form edible fruiting bodies that are of high economic value [8,9].

The development of ECM is a dynamic process, requiring elaborately regulated interactions between plant roots and compatible fungi. First, the host plant and fungus recognize each other by releasing and receiving signals, which induce lateral root formation, fungal spore germination, and mycelial branching. These changes increase the chances of ECM fungal hyphae encountering plant roots [10,11,12,13,14,15,16,17,18]. Next, the mycelium attaches to the root and starts to colonize. The mycelium stretches along the root surface and the intercellular space, and eventually differentiates to form a mantle and Hartig net [1,19]. In general, most ECM associations can be characterized by both mantle and Hartig net structures, and the formation of Hartig net is defined as signs of functional ECM establishment, which is considered functioning in nutrient exchanges between the two partners [20,21]. However, in some ECM associations formed between ascomycetes and broadleaf trees, the mantle may be poorly developed or essentially non-existent [1].

ECM formation is accompanied by changes in gene expression [16,19,22,23,24], involving cell growth and differentiation, signaling, defense, energy production, and other functional genes [21,22,25,26,27,28,29,30,31,32,33,34,35,36,37,38,39,40]. The expression patterns of these genes follow a complex series of sequential steps [24,27]. Small secretory proteins induced by mycorrhizae, such as MiSSP7 [41,42,43], MiSSP7.6 [44], MiSSP8 [45], and MiSSP10b [46], help fungi evade host defenses and play an important role during the early stages of ECM formation. Genes associated with symbiosis-induced malate synthase [47], arginine methyltransferase [33], endoglucanase (*LbGH5-CBM1*) [48], polygalacturonase (*LbGH28A*) [49], and pectin methylesterases [21] are involved during the late stages of ECM development. A phosphorus transporter (*HcPT2*) [50] and an ammonium transporter (*AMT2.2*) [51,52] play important roles in maintaining the function of the ECM when functional ECM structures—the mantle and Hartig net—are formed. These findings have greatly contributed to our understanding of the molecular mechanism of ECM symbiosis. However, these previous findings have been limited to a few specific ECM combinations, such as *Populus*-*Laccaria bicolor* [21,41], *Betula*-*Paxillus involutus* [25,27], *Eucalyptus*-*Pisolithus tinctorius/Pisolithus microcarpus* [21,26], and *Pinus pinaster*-*Hebeloma cylindrosporum* [32]. Given the huge numbers of fungi and tree species that can form ECM and the complexity of these relationships, the establishment process of different ECM combinations is likely to be specific [53]. Therefore, it is necessary to expand our studies to other ECM combinations to gain a comprehensive understanding of this important symbiotic association. Another problem is that there are little data on the molecular regulation of the ECM symbiotic process because previous studies have mainly focused on specific stages (usually the functional stage) of the ECM formation process.

*Pinus massoniana* is one of the main timbers and a pioneer afforestation tree species in China; however, the survival and breed of this unique native tree species are highly dependent on ECM fungi [54,55,56,57]. Our group previously reported that *Suillus bovinus* is the dominant ECM fungus in *P. massoniana* forests. In addition, *S. bovinus* produces edible fruit bodies with a high economic value [58]. As well as other species in the genus of *Suillus*, *S. bovinus* present a high degree of host specificity towards conifers, and its distribution coincides with the natural distribution of Pinaceae in the Northern Hemisphere [59,60]. *Suillus* species are also recognized as the earliest colonizers of pine seedlings which play vital roles in conifer invasions [61,62]. Although we have a good understanding of the function of the *P. massoniana* ECM, the mechanisms involved in the formation of the ECM remain unclear, particularly at the transcriptional level. In this study, we investigated the morphological and transcriptional characteristics of *P. massoniana* roots when inoculated with *S. bovinus*. We clarified the stages of the formation process of this ECM association by morphological profiling and then performed transcriptional profiling. Both transcriptional and physiological data suggest that the phenylpropanoid biosynthesis pathway may play important roles in ECM formation.

## 2. Materials and Methods

### 2.1. Plant and Fungal Materials

Seeds of *P. massoniana* (collected from the *P. massoniana* national base at Maanshan Forest Farm, Duyun City, Guizhou Province, China) were cleaned with 0.01% Tween 20, and then sterilized, first with 0.5% KMnO_4_ solution for 2 h, and finally with 0.01% Tween 20 and 0.5% carbendazim for 1 h, and then with antibiotics—200 mg/L streptomycin and 100 mg/L gentamicin for 20 min. At the end of each of these steps, the seeds were cleaned with sterile water three times for 5 min each time. The sterilized seeds were then placed in wet vermiculite and incubated in 25 °C climate chambers with 14 h of light (150 μmol m^−2^s^−1^) and 10 h of darkness (light 0 μmol m^−2^s^−1^) per day. Thirty-day-old *P. massoniana* seedlings were used in the ECM formation trials.

Seeds of wild-type *Arabidopsis thaliana* Columbia-0 (kindly provided by Prof. Fuhua Fan) were sterilized with 75% alcohol for 1 min and with 5% NaClO solution for 10 min. At the end of each of these steps, the seeds were cleaned with sterile water three times for 5 min each time. Seeds were then transferred to Murashige and Skoog medium and incubated in 25 °C climate chambers with 14 h of light per day (150 μmol m^−2^s^−1^) and 10 h of darkness.

The strain *S. bovinus* LL-1 was isolated from a fruiting body collected in a *P. massoniana* forest [52]. The mycelium of *S. bovinus* was subcultured and maintained on modified Melin-Norkran’s (MMN) [63] medium at 25 °C in the dark. The composition of MMN medium is 25 mg/L NaCl; 250 mg/L (NH_4_)_2_HPO_4_; 500 mg/L KH_2_PO_4_; 5 mg/L FeCl_3_; 50 mg/L CaCl_2_; 150 mg/L MgSO_4_·7H_2_O; 100 mg/L VB1; 10 g/L glucose; 1.00 g/L casamino acids; 5.00 g/L malt; and 10 g/L agar.

### 2.2. In Vitro Mycorrhizal Formation between P. massoniana and S. bovinus

To investigate the morphological features of the pre-symbiotic phase (before physical contact has been made between the host and the fungus), we carried out two trials to test the effects of volatile organic compounds (VOCs) and exudates released by *S. bovinus* on *P. massoniana* growth.

(a) VOC effects: 30 mL of basal medium (DCR) (composed of 400 mg/L NH_4_NO_3_; 556 mg/L Ca(NO_3_)_2_·4H_2_O; 370 mg/L MgSO_4_·7H_2_O; 85 mg/L CaCl_2_·2H_2_O; 170 mg/L KH_2_PO_4_; 6.2 mg/L H_3_BO_3_; 22.3 mg/L MnSO_4_·H_2_O; 8.6 mg/L ZnSO_4_·7H_2_O; 0.25 mg/L CuSO_4_·5H_2_O; 0.83 mg/L KI; 0.025 mg/L CoCl_2_·6H_2_O; 0.025 mg/L LiCl; 0.25 mg/L NaMoO_4_·2H_2_O; 27.8 mg/L FeSO_4_·7H_2_O; 37.3 mg/L EDTA-2Na; 1.0 mg/L VB_1_; 0.5 mg/L VB_6_; 0.5 mg/L nicotinic acid; 2.0 mg/L glycine; 200 mg/L myo-inositol; 10 g/L sucrose; and 10 g/L agar) [64] was poured into 13 cm × 13 cm Petri dishes. Once the medium had solidified, the medium in one half of each Petri dish was cut out with a scalpel, and then 15 mL of MMN medium was poured into the empty half. Once the MMN medium had solidified, a 2 cm × 13 cm strip of medium at the junction of the two media was cut with a scalpel and removed to leave a 2 cm gap between the two 5 cm × 13 cm blocks of media in each dish. The MMN medium was inoculated with an *S. bovinus* plug (1 cm in diameter) and a *P. massoniana* seedling was transplanted onto the DCR medium (VOC treatment). Plates that were not inoculated with *S. bovinus* were considered to be controls (NVOC treatment).

(b) Exudate effects: a *P. massoniana* seedling was transplanted onto DCR medium (30 mL) in 13 cm × 13 cm Petri dishes. A cellophane membrane was placed over the roots and then covered with a thin layer of MMN medium. The MNM medium was then inoculated with an *S. bovinus* plug (1 cm in diameter) (Exud treatment). Plates that were not inoculated with *S. bovinus* were considered to be controls (NExud treatment).

To verify whether the effects of *S. bovinus* VOCs and exudates are host-specific, we also set up plates to observe the effects of *S. bovinus* VOCs and exudates on the root growth of the non-host *A. thaliana* using the same experimental procedures as those described above.

To investigate the morphological features of the symbiotic phase, a *P. massoniana* seedling was transplanted onto DCR medium (30 mL) in 13 cm × 13 cm Petri dishes and an *S. bovinus* plug (1 cm in diameter) was placed in direct contact with the *P. massoniana* taproot (M treatment). *P. massoniana* taproots that were not inoculated with an *S. bovinus* plug were considered to be controls (NM treatment). There were 20 replicates of each treatment. The bottom of each Petri dish was wrapped with tinfoil to cover the root growth area before placing the Petri dish vertically in a climate chamber at 25 °C with 14 h of light (light 120 μmol m^−2^s^−1^) and 10 h of darkness (light 0 μmol m^−2^s^−1^) per day.

### 2.3. Morphological Observations

Morphological observations at the pre-symbiosis phase were recorded by scanning Petri dishes (Epson Perfection V330 Photo) every 7 days. Root length and branch measurements were obtained using the ImageJ SmartRoot plug-in. However, because it was difficult to observe the number of *Arabidopsis* root branches in the Petri dish, the number of root branches was only recorded on the day of harvest at 28 days post-inoculation (dpi).

To characterize the morphology at the symbiosis stage, five seedlings were randomly selected for observation every 7 days from inoculation with *S. bovinus* until ECM formation. First, the contact between hyphae and roots was examined under a stereomicroscope (M205FA, Leica Microsystems, Wetzlar, Germany). Second, transversal cross-sections of at least 20 independent root segments were cleared (5% KOH solution, 90 °C for 2 h) to make them transparent, acidified (1% HCl solution *w*/*v*, 10 min at room temperature), and then stained with 0.03% chlorazol black (90 °C for 20 min). The stained sections were then mounted in glycerol and observed under a light microscope (DM3000, Leica Microsystems, Wetzlar, Germany).

### 2.4. RNA Extraction, Sequencing and Analysis

Given that the exudate-induced effects were host-specific, whereas the VOC-induced effects were not, plants treated with exudates were selected as materials for transcription analysis at the pre-symbiotic stage. Transcriptome profiling was conducted on *P. massoniana* roots treated with *S. bovinus* exudates for 14 days and on *P. massoniana* roots 7 dpi (inoculated M7, uninoculated NM7) and 28 dpi (inoculated M28, uninoculated NM28) with *S. bovinus*. For each treatment, four biological replicates were collected. The samples were frozen in liquid nitrogen and stored at −80 °C until RNA extraction.

Total RNA was extracted using an RNAprep Pure Plant Kit (TIANGEN, Beijing, China). Samples were first subjected to quality control using an Agilent 2100 Bioanalyzer (Agilent Technologies, Santa Clara, CA, USA) and then sent to Novogene (Beijing, China) for sequencing on the Illumina HiSeqTM2000 platform.

Raw data obtained by sequencing included a small number of reads with a sequencing adapter or that were of low sequencing quality. To ensure the quality and reliability of data analysis, the original data were filtered as follows to remove: (1) reads containing an adapter; (2) reads containing N (N indicates that base information cannot be determined); (3) low-quality reads (a Qphred score of ≤20 bases for more than 50% of the total read length).

### 2.5. Determination of Differentially Expressed Genes and Enrichment Analysis

Differentially expressed genes (DEGs) were analyzed with DESeq2 [65], and genes with an absolute log_2_-fold change value of ≥1 and an adjusted *p*-value of <0.05 were deemed to be differentially expressed. Gene Ontology (GO) enrichment analysis was performed based on the GOseq method [66], and Kyoto Encyclopedia of Genes and Genomes (KEGG) enrichment analysis was performed using KOBAS (2.0) [67].

RNA-seq data for all samples are available at the Sequence Read Archive of the National Center for Biotechnology Information (http://www.ncbi.nlm.nih.gov/sra, accessed on 24 October 2022) under accession number PRJNA886481.

### 2.6. Effect of Acibenzolar Acid on Mycorrhizal Development

Hydroxycinnamoyl-CoA shikimate/quinate transferase (HCT) plays an important role in lignin synthesis; however, acibenzolar acid has an inhibitory effect on this enzyme [68]. We analyzed the effect of acibenzolar acid on HCT activity and mycorrhizal development in *P. massoniana*.

Acibenzolar acid (CAS: 35272-27-6; Dr. Ehrensorfer GmbH, Augsburg, Germany) dissolved in ethanol and filtered through a 0.22 μm diameter aperture was added to the DCR medium at concentrations of 0, 100, 300, or 500 μM. After four weeks of colonization, mycorrhizal development was examined according to the method described above. The ImageJ platform was used to measure the Hartig net depth and area (the area of fungal hyphae between cells). Five micrographs were measured for each treatment and three measurements were recorded per micrograph.

The HCT activity of *P. massoniana* roots was analyzed using an HCT ELISA detection kit (JingMei, JiangSu, China; www.jsjmsw.com, accessed on 17 October 2022) according to the manufacturer’s instructions.

### 2.7. Statistical Analysis

Apart from the DEG analysis data, data were analyzed using SPSS 25.0 software (IBM^®^ SPSS^®^ Statistics). The effects of VOCs and exudates on root growth (root length and number of root branches) were analyzed using one-way analysis of variance (ANOVA). A Student’s *t*-test was used to determine significant differences between means. ANOVA of *P. massoniana* HCT activity under different treatments, the Hartig net depth, and area were analyzed by performing a Duncan’s test. The principal component analysis (PCA) of the DEGs was performed using the OmicShare tools (https://www.omicshare.com/tools, accessed on 29 October 2022).

## 3. Results

### 3.1. Pre-Symbiotic Interactions between P. massoniana and S. bovinus

**Figure 1:**
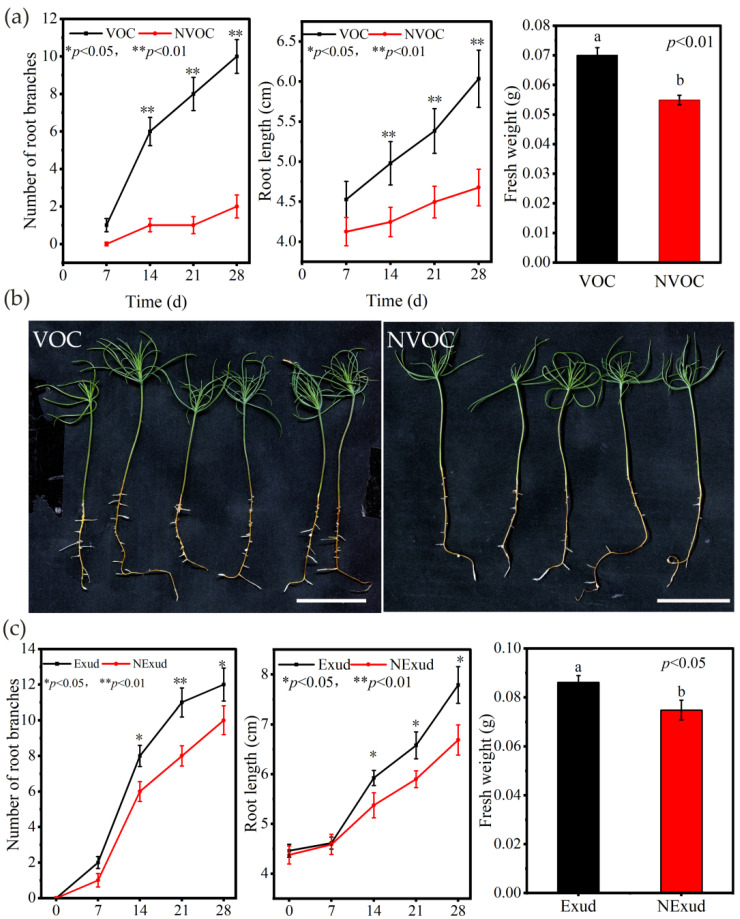

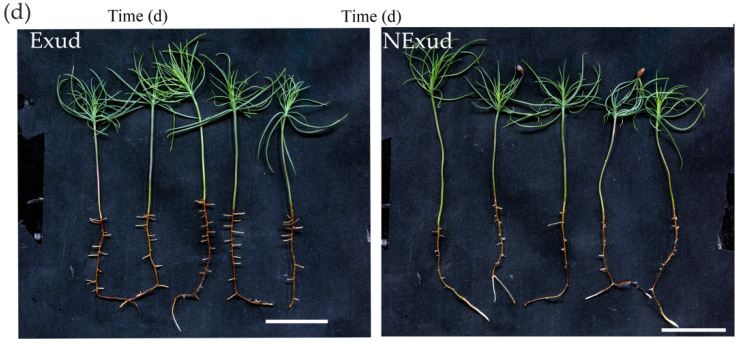
Effects of volatile organic compounds (VOCs) and exudates released by *Suillus bovinus* on the growth of *Pinus massoniana*. (**a**) Number of root branches, root length, and fresh weight of *P. massoniana* seedlings treated with VOCs and without VOCs (NVOC), *n* = 15; (**b**) *P. massoniana* seedlings subjected to VOC (28 d) and NVOC (28 d) treatments, scale bars = 2 cm; (**c**) root branches, root length, and fresh weight of *P. massoniana* seedlings subjected to the exudate (Exud) (28 d) and non-exudate (NExud) (28 d) treatments, *n* = 20; (**d**) *P. massoniana* seedlings subjected to Exud (28 d) and NExud (28 d) treatments, scale bars = 2 and 3 cm. ** *p* < 0.01; * *p* < 0.05. Bars represent mean values ± the SE. Different letters above bars indicate significant differences between treatments.

VOCs and exudates released by *S. bovinus* significantly promoted the root length and number of root branches of *P. massoniana* from the 14th day of treatment compared with control treatments (Figure 1). In addition, VOCs and exudates significantly increased the biomass of pine seedlings by 29.37% and 15.07%, respectively, compared with the NVOC and NExud control treatments (Figure 1a,c).

**Figure 2:**
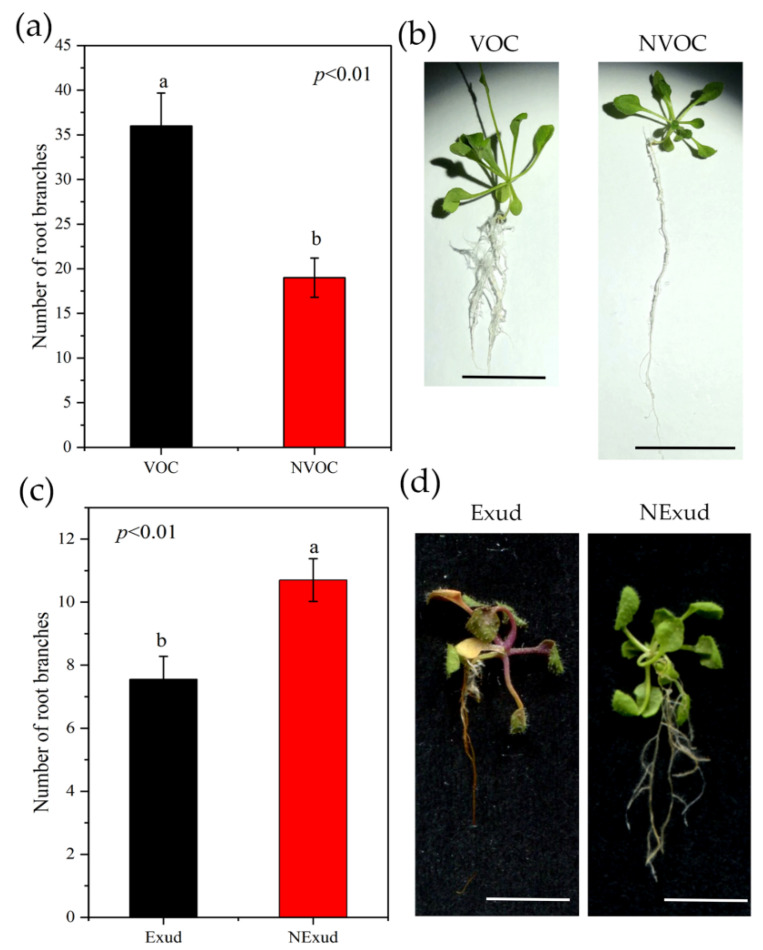
Effects of VOCs and exudates released by *S. bovinus* on the growth of *Arabidopsis thaliana*. The effect of VOCs on: (**a**) *A. thaliana* root branch number, *n* = 15; (**b**) *A. thaliana* plants, scale bars = 2 cm. Effects of *S. bovinus* exudates on: (**c**) *A. thaliana* root branch number, *n* = 20; (**d**) *A. thaliana* plants, scale bars = 2 and 3 cm. Bars represent mean values ± the SE. Different letters above bars indicate significant differences between treatments.

However, *S. bovinus* VOCs and exudates had the opposite effects on *A. thaliana* growth. Although *S. bovinus* VOCs promoted *A. thaliana* root branching, they inhibited the elongation of the taproot (Figure 2a,b), and *S. bovinus* exudates inhibited both the shoot and root growth of *A. thaliana* (Figure 2c,d).

### 3.2. Symbiotic Interactions between P. massoniana Roots and S. bovinus over Time

**Figure 3:**
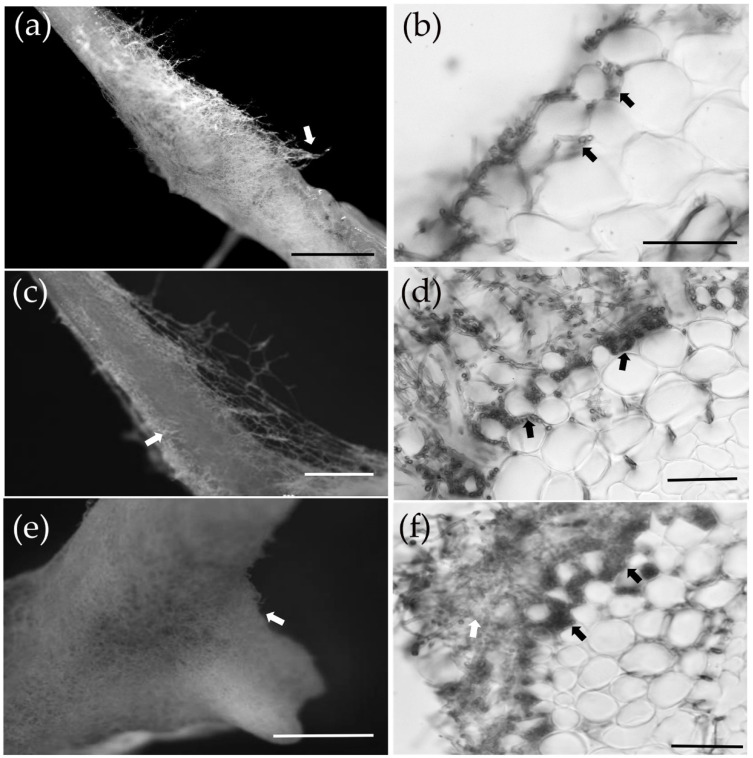

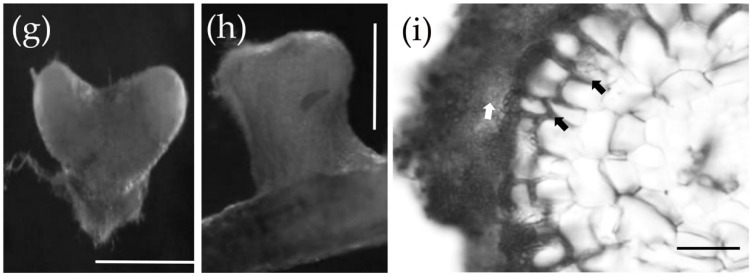
The ectomycorrhizal (ECM) formation process between *P. massoniana* and *S. bovinus*. (**a**,**b**) At 7 days post-inoculation (dpi): scale bars = 5 mm and 500 µm, respectively. The white arrow indicates hyphae wrapped around the root system; black arrows indicate the intercellular mycelium. (**c**,**d**) At 14 dpi: scale bars = 1 mm and 100 µm, respectively. The white arrow indicates hyphae wrapped around the root system; black arrows indicate the developing Hartig net. (**e**,**f**) At 21 dpi: scale bars = 1 mm and 50 µm, respectively. The white arrow in (**e**) indicates hyphae wrapped around the root system. The white arrow in (**f**) indicates the developing mantle and black arrows indicate the Hartig net. (**g**–**i**) At 28 dpi: scale bars = 1 mm, 500 μm, and 50 μm, respectively. (**g**) Dichotomous branching hypha, (**h**) swollen hypha. (**i**) Cross-section of the root: the white arrow indicates the mantle and black arrows indicate the Hartig net.

At 7 dpi, the mycelium of *S. bovinus* had proliferated on the surface of *P. massoniana* roots and started to invade the intercellular space (the invasion stage) (Figure 3a,b). By 14 dpi, more hyphae had aggregated around the root surface and grown into inter-radical spaces (Figure 3c,d). By 21 dpi, hyphae had covered the root surface to form a mantle-like structure (Figure 3e), and hyphae had penetrated the root intercellularly to form a Hartig net structure (a Hartig net was considered to have formed when hyphae had invaded the root system and completely wrapped one to two layers of cortical cells, Figure 3f). A functional mycorrhiza had established by 28 dpi, at which point the Hartig net and mantle (a mantle was considered to have formed when more than four layers of hyphae were tightly wrapped around a root; Figure 3i) were fully developed, and the root tips showed dichotomous branching and were swollen in shape (mantle and Hartig net formation stage) (Figure 3g,h).

### 3.3. Root Transcriptional Analysis during Symbiotic Interactions between P. massoniana and S. bovinus

#### 3.3.1. Quality Analysis

Transcriptome sequencing analysis of all *P. massoniana* root samples resulted in mean raw reads of 40,419,484 and 35,330,493 for the Exud and NExud treatments, respectively; 70,190,257 and 68,570,981 for the M7 and NM7 treatments, respectively; and 41,427,706 and 35,802,254 for the M28 and NM28 treatments, respectively. By removing reads with adapters and low-quality reads from raw reads, mean clean reads of 38,892,360 and 34,386,741 were obtained for the Exud and NExud treatments, 68,939,003 and 67,174,335 for M7 and NM7 treatments, respectively, and 40,267,781 and 34,747,055 for the M28 and NM28 treatments, respectively. The Q20 and Q30 of all samples were above 97% and 92%, respectively, and the GC content was also at a normal level (Table S1).

#### 3.3.2. Analysis of DEGs

**Figure 4:**
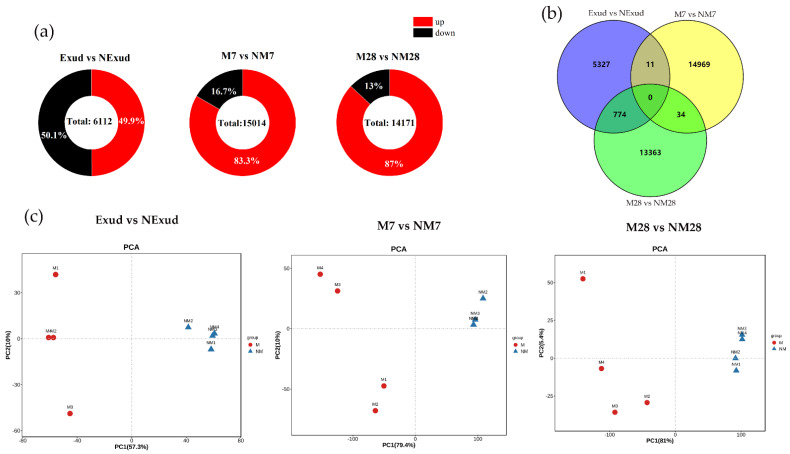
Number of differentially expressed genes (DEGs). (**a**) Pie charts showing the number of significantly upregulated and downregulated DEGs at each timepoint (*p* < 0.05); (**b**) Venn diagram showing the number of DEGs that are common to more than one timepoint and the number of genes that are unique to individual timepoints; (**c**) principle component analysis (PCA) of DEGs detected in the Exud vs. NExud treatment, 7 dpi with *S. bovinus* (M7) vs. the uninoculated (NM7) treatment, and 28 dpi with *S. bovinus* (M28) vs. the uninoculated (NM28) treatment.

In total, across the three colonization time points there were 35,297 DEGs due to *S. bovinus* colonization. The lowest number of DEGs was detected during the pre-symbiotic phase, when the plant and fungus were separated physically by a cellophane membrane. There were nearly equal numbers of upregulated and downregulated genes at this time point (Figure 4a). The largest number of DEGs was found at the invasion stage (7 dpi), followed by the Hartig net and mantle formation stage (28 dpi). In addition, 11 DEGs were co-expressed at the pre-symbiotic stage and the invasion stage, while at the symbiosis stage, 34 DEGs were co-expressed at the invasion and the Hartig net and mantle formation stage (Figure 4b). PCA showed that DEGs in samples from inoculated and non-inoculated treatments at the three stages of symbiosis were significantly separated (Figure 4c).

#### 3.3.3. Enrichment Analysis of DEGs

**Figure 5:**
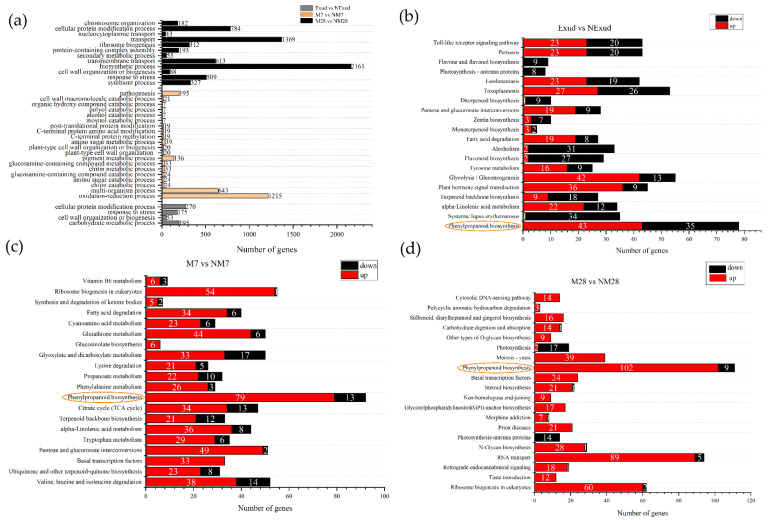
Enrichment of DEGs. (**a**) Enriched Gene Ontology biological processes. (**b**–**d**) Kyoto Encyclopedia of Genes and Genome enrichment for Exud vs. NExud, M7 vs. NM7, and M28 vs. NM28. The numbers shown on the red and black bars indicate the number of upregulated and downregulated DEGs, respectively. The phenylpropanoid biosynthesis pathway (circled) was common to all three symbiosis stages.

GO was used to functionally classify DEGs during *P. massoniana–S. bovinus* ECM formation. Here, we focused on the enrichment of DEGs in the biological process category. We found 693 DEGs at the pre-symbiotic stage, 2493 at 7 dpi, and 6636 at 28 dpi (Figure 5a). At the pre-symbiotic stage, the top two terms were “cellular protein modification process” and “carbohydrate metabolic process”; at 7 dpi, the top two terms were “oxidation–reduction process” and “multi-organism process”; and at 28 dpi, the top two terms were “biosynthetic process” and “transport”. In addition, we found that DEGs associated with cell walls were significantly enriched during all three stages.

These DEGs were further analyzed using KEGG to fully explore their functions. At the pre-symbiotic stage, most DEGs were mapped to the biosynthesis of other secondary metabolites, carbohydrate and lipid metabolism, signal transduction, and the immune system, e.g., phenylpropanoid biosynthesis (78; ko00940), glycolysis/gluconeogenesis (55; ko00010), plant hormone signal transduction (45; ko04075), the Toll-like receptor signaling pathway (43; ko04620), and alpha-linolenic acid metabolism (34; ko00592), and the number of upregulated and downregulated DEGs was similar (Figure 5b). At 7 days of symbiosis, a number of DEGs were mapped to the biosynthesis of secondary metabolites, genetic information processing, amino acids, and carbohydrate metabolism, e.g., phenylpropanoid biosynthesis (92), ribosome biogenesis in eukaryotes (55; ko03008), valine, leucine, and isoleucine degradation (52; ko00280), pentose and glucuronate interconversions (51; ko00040) and glyoxylate and dicarboxylate metabolism (50; ko00630), and most of these DEGs were upregulated (Figure 5c). At 28 days of symbiosis, functional ECM had formed. Most DEGs were involved in the biosynthesis of other secondary metabolites, genetic information processing, and glycan biosynthesis and metabolism, e.g., phenylpropanoid biosynthesis (111), RNA transport (94; ko03013), ribosome biogenesis in eukaryotes (62), *N*-glycan biosynthesis (29; ko00510) and basal transcription factors (24; ko03022), and most of these DEGs were upregulated (Figure 5d). Phenylpropanoid biosynthesis was a common pathway in ECM development processes in *P. massoniana* and *S. bovinus* (Figure 5b–d). Furthermore, the pathway with the most DEGs at each stage was the phenylpropanoid biosynthesis pathway. The number of DEGs associated with the phenylpropanoid biosynthesis pathway increased over time and their expression patterns differed at different stages of symbiosis. The phenylpropanoid biosynthesis pathway exhibited a mixture of both positive and negative gene regulation during the pre-symbiotic stage. In the invasion and functional stages, most genes involved were generally upregulated. Moreover, as the ECM formation process progressed, the number of upregulated DEGs in this pathway gradually increased, while the number of downregulated DEGs decreased.

#### 3.3.4. Genes Involved in the Phenylpropanoid Biosynthesis Pathway throughout Colonization

**Figure 6:**
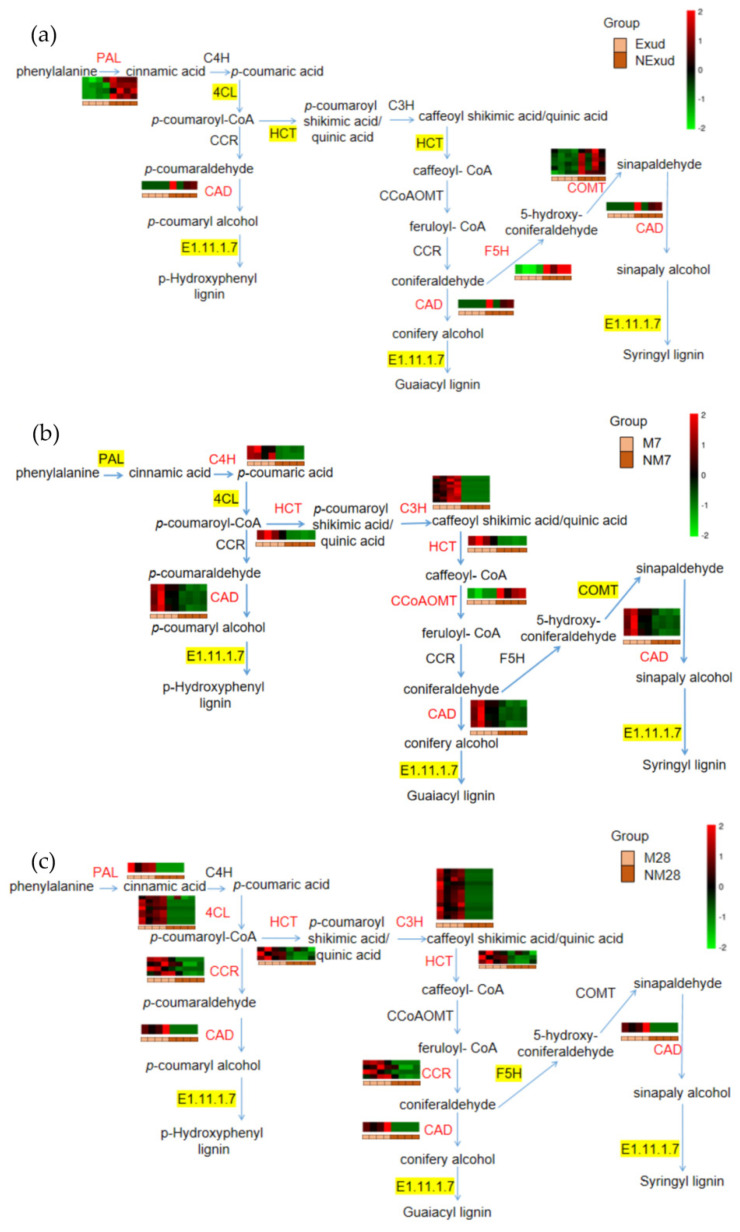
Phenylpropanoid biosynthesis pathway with heatmaps showing fold changes in the expression of genes encoding key enzymes in this pathway. (**a**) Exud vs. NExud; (**b**) M7 vs. NM7; (**c**) M28 vs. NM28. Key enzymes encoded by DEGs that were annotated in metabolic pathways are shown in red. An enzyme highlighted in yellow indicates that both upregulated and downregulated genes encode the enzyme. An enzyme that is not highlighted in yellow indicates that no genes encoding the enzyme were annotated. Enzymes: PAL, phenylalanine ammonia-lyase; C4H, cinnamate 4-hydroxylase; 4CL, 4-coumarate:CoA ligase; HCT, hydroxycinnamoyl-CoA shikimate/quinate transferase; C3H, coumarate 3-hydroxylase; CCoAOMT, caffeoyl-CoA-*O*-methyltransferase; CCR, cinnamoyl CoA reductase; F5H, ferulate 5-hydroxylase; COMT, caffeic acid *O*-methyltransferase; CAD, cinnamyl alcohol dehydrogenase.

In this study, phenylpropanoid biosynthesis was found to be a common pathway and to have the largest number of DEGs that were shared at different stages of the symbiotic process during the formation of the ECM between *P. massoniana* and *S. bovinus*. We focused our attention on the lignin synthesis process of this pathway. The genes encoding phenylalanine ammonia-lyase (PAL) and cinnamyl alcohol dehydrogenase (CAD), which are involved in lignin synthesis, were significantly expressed during all three phases of ECM formation. We also found that genes involved in PAL synthesis had similar expression patterns to those of genes encoding CAD during the pre-symbiotic and mantle and Hartig net formation stages. Prior to physical contact, PAL genes (*Cluster*-*21925.46012*, *Cluster*-*21925.78638*, *Cluster*-*21925.44510*, *Cluster*-*21925.71279*) and the CAD gene (*Cluster*-*21925.8529*) were downregulated as a result of the *S. bovinus* exudate treatment (Figure 6a). However, at the Hartig net and mantle formation stage, PAL (*Cluster*-*21925.21162*) and CAD genes (*Cluster*-*9078.0*) were upregulated (Figure 6c). At the invasion stage, the expression patterns of the genes encoding PAL were mixed and the CAD gene was upregulated (*Cluster*-*20437.23132*, *Cluster*-*20437.24246*, *Cluster*-*20437.24240*, *Cluster*-*20437.24241*) (Figure 6b). Furthermore, the genes encoding HCT were not significantly expressed during the pre-symbiotic stage; however, at the symbiotic stage, they were generally upregulated; coumarate 3-hydroxylase (C3H) also showed the same expression pattern (Figure 6, Table S2).

### 3.4. Effect of Inhibiting HCT Activity on ECM Morphogenesis

**Figure 7:**
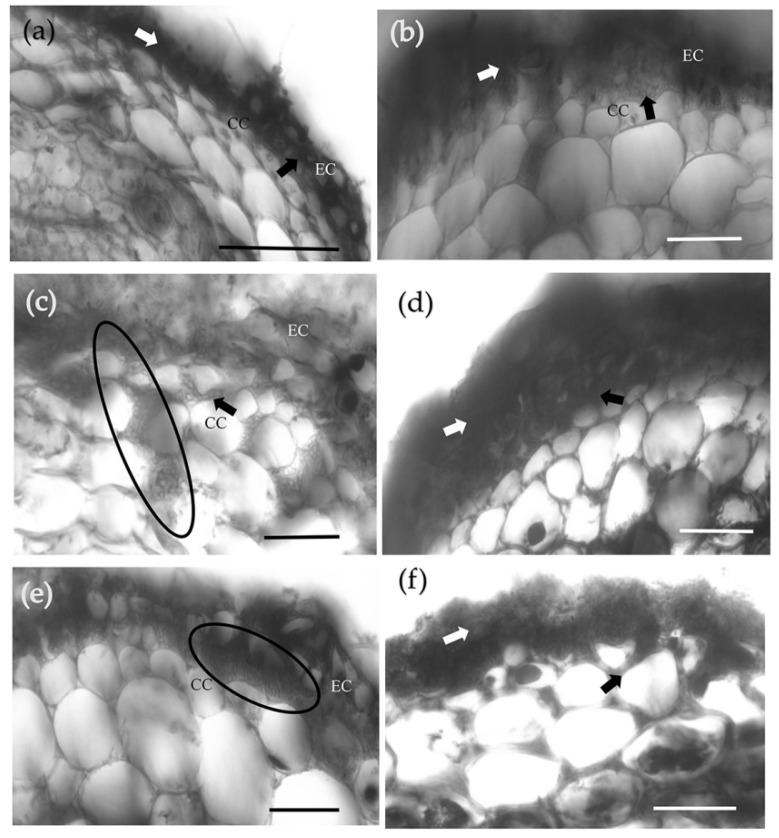
Effects of acibenzolar acid treatment on ECM development. Transverse cross-sections of a *P. massoniana* lateral root colonized by *S. bovinus*: (**a**) without the addition of acibenzolar acid; (**b**) in the presence of 100 μM acibenzolar acid; (**c**,**d**) 300 μM acibenzolar acid; and (**e**,**f**) 500 μM acibenzolar acid. Scale bars = 50 µm. Black arrows and ellipses indicate Hartig nets, white arrows indicate the mantle. Abbreviations: CC, cortical cells; EC, epidermal cells.

**Figure 8:**
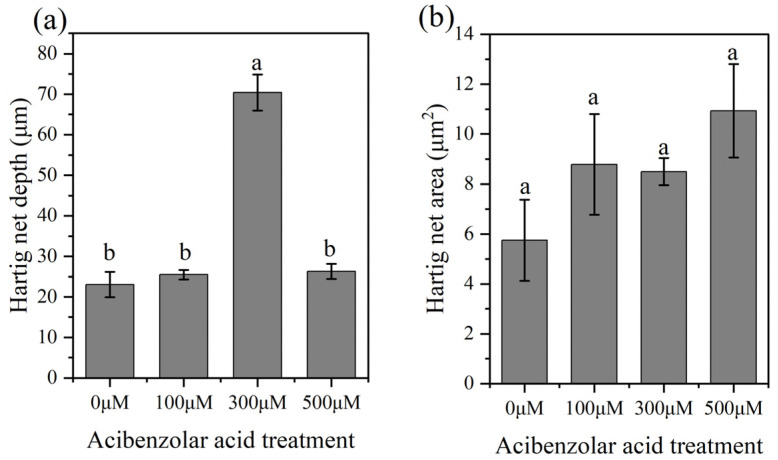
Effects of acibenzolar acid treatment on Hartig net development. (**a**) Hartig net depth; (**b**) Hartig net area. Bars represent means ± the SE, *n* = 5; different letters above bars indicate significant differences between treatments at *p* < 0.05.

HCT enzyme activity was significantly inhibited by the presence of 300 μM acibenzolar acid (Figure S1), and the development of Hartig nets was further promoted. Following the 300 μM acibenzolar acid treatment, hyphae penetrated the intercellular spaces of the third layer of cortical cells (Figure 7c,d), layers 1–2 at 100 μM (Figure 7b), and layers 2–3 at 500 μM (Figure 7e,f). In contrast, in the absence of acibenzolar acid, mycelia only encased the first layer of cortical cells, and most of the mycelia spread intercellularly between epidermal cells to form a labyrinthine structure (Figure 7a). A significantly deeper Hartig net developed following the 300 μM treatment (70.4 ± 4.43 μm; *p* < 0.05), compared with the 500 μM (26.33 ± 1.86 μm), 100 μM (25.49 ± 1.16 μm) or 0 μM (23.08 ± 3.12 μm) acibenzolar acid treatments (Figure 8a). The presence of acibenzolar acid also affected the Hartig net area intercellular spaces (500 μM > 100 μM > 300 μM > 0 μM); however, these data were not statistically significant (Figure 8b).

## 4. Discussion

An ECM association between *P. massoniana* and *S. bovinus* is typical in forests of south China. In this study, we focused on the morphological and transcriptional changes that occurred over time during the formation of this ECM symbiotic association. Our findings showed that during the formation of the ECM between *P. massoniana* and *S. bovinus*, the symbiotic morphogenesis was coordinated with root transcriptional adjustments.

The establishment of a mycorrhizal association between *P. massoniana* and *S. bovinus* can be divided into two stages: the pre-symbiotic stage (signal recognition before physical contact) and the symbiosis stage. Studies have shown that before physical contact, mycorrhizal fungi can affect host root development by releasing signaling molecules (including VOCs and exudates) to promote, for example, root elongation and branching [13,14,15,69,70,71], so as to increase the contact opportunities between fungi and host roots [1]. We also found that both VOCs and exudates released by *S. bovinus* stimulated *P. massoniana* growth and root branching. However, the exudate-induced effects showed host plant specificity, whereas VOC-induced effects did not. Previous studies have also reported that ECM fungal volatiles can promote *A. thaliana* root growth [13,15,69,70,71]. Compared with exudates, VOCs have long-distance diffusion features. We speculate that ECM fungi stimulate plant root growth from a long distance, and when ECM fungi and plant roots come within a certain distance of each other, exudate-induced effects further help ECM fungi to discriminate potential host plants from non-host plants.

Fungi and hosts enter the symbiotic stage after successful mutual recognition. The mycelium gathered around the root surface develops into a mantle, and the intraradical mycelium invades the intercellular space among cortical cells to form a Hartig net [1,72,73]. In this study, we found that at 7 dpi, mycelium began to invade the intercellular space among root cortical cells, and by 28 dpi, the mantle and Hartig net had developed and matured. The time required for ECM formation differs in different studies, ranging from four days to two weeks, or up to one month [26,74,75,76,77,78]. These differences may be due to the species of ECM fungus involved and the host specificity. Differences in the time required for ECM formation have also been observed between strains, even when they are forming an ECM with the same host. Yu (2007) [78] observed that different strains of *Cortinarius* sp. and *Picea koraiensis* formed ECM at different rates, ranging from 21 days to more than one month. Moreover, the experimental system and culture environment may also affect the time needed for mycorrhizal formation. Opinions about the development sequence of the Hartig net and mantle also vary. Some studies have reported that the Hartig net forms before the mantle [53,79,80,81], while others have suggested the opposite [26,69,74,82,83]. These different conclusions may be due to the specificities of the ECM formation process between distinct ECM fungal species and host plant combinations (particularly gymnosperms and angiosperms). In this study, the Hartig net and mantle developed synchronously during the ECM formation between *P. massoniana* and *S. bovinus*; however, the Hartig net formed earlier than the mantle.

ECM formation is a dynamic process, and all morphological changes involve coordinated changes in gene expression [22,25,28,29,30,36]. Our data showed that most DEGs were present at the invasion stage (7 dpi), indicating that *P. massoniana* seedlings underwent a greater range of transcriptional reprogramming at the beginning of the symbiotic stage in response to *S. bovinus* infection. Moreover, there were no common DEGs at the three stages of symbiosis, suggesting that there may be a unique set of molecular mechanisms supporting the formation of a symbiotic association between *P. massoniana* and *S. bovinus*. Using GO enrichment analysis, we focused on those biological processes that were most significantly enriched and found that some DEGs at each stage were significantly enriched in categories relating to cell structure. Given that the invasion of ECM fungi can cause a significant loosening of plant root cells [84,85], these DEGs may play an important role in ECM symbiosis. KEGG enrichment was used to further analyze the function of DEGs. During the pre-symbiotic stage, the main task of the host and fungus is to recognize each other by releasing signals [16,17,18,19], which include many secondary metabolites and plant hormones [11,12,13,15,76,84]. In this study, we found that during this stage a large number of DEGs were associated with the biosynthesis of secondary metabolites and signal transduction. Successful colonization by fungi often induces the breakdown of host carbohydrate to meet the autogenous growth needs [1]. Such a reprogramming of carbohydrate metabolism induced by *S. bovinus* may be initiated prior to physical contact because a large proportion of DEGs were associated with carbohydrate metabolism during the pre-symbiotic stage. These metabolic pathway changes lay the foundation for successful colonization and symbiosis later on. At the infection phase (7 dpi), in addition to DEGs involved in the biosynthesis of secondary metabolites and carbohydrate metabolism, a large number of DEGs were associated with genetic information processing and amino acid metabolism, and most of these DEGs were upregulated. Amino acids are important sinks for carbon assimilation [86], particularly aliphatic amino acids such as valine, leucine, and isoleucine. Thus, genes involved in the degradation of these amino acids may provide carbon sources for *S. bovinus*. Once a functional ECM was established (28 dpi), the DEGs were mainly associated with RNA transport, ribosome biogenesis in eukaryotes, basal transcription factors, and the cytosolic DNA-sensing pathway, which may further facilitate substance exchanges between the two symbionts [76].

Phenylpropanoid biosynthesis was a common pathway during the development of this ECM symbiosis. Related DEGs showed a mixture of both up-and downregulated expression patterns at the pre-symbiotic stage; however, related DEGs were upregulated at 7 dpi and 28 dpi. Different studies have reported different findings regarding the expression patterns of DEGs involved in this pathway. During the early stages of ECM colonization, Weiss et al. [87] and Plett et al. [88] reported that phenylpropanoids were increased; however, Hill et al. [77] suggested that this pathway was downregulated during the invasion phase and upregulated during the functional symbiosis phase. Some studies have shown that the colonization of ECM could induce phenylpropanoid metabolism in hosts and hinder the colonization process [89,90]. For example, certain metabolites produced by this pathway can limit hyphal penetration and the formation of the Hartig net [90]. However, Behr et al. [91] found that transgenic lines with downregulated genes in the phenylpropanoid pathway all showed lower colonization rates compared with the wild type, indicating that metabolite production in this pathway favors mycorrhizal formation. The reason for these two different conclusions may be that colonization by different ECM fungi results in different end products being produced by the host phenylpropane biosynthesis pathway.

HCT is a rate-limiting enzyme of phenylpropanoid biosynthesis and is important for the products synthesized by the phenylpropane biosynthesis pathway [92]. According to our data, at both 7 and 28 dpi, the colonization of *S. bovinus* significantly induced the expression of HCT genes in *P. massoniana* roots. To gain more information about the function of HCT, we investigated the effects of the activity of HCT on ECM formation between *S. bovinus* and *P. massoniana* by using a specific enzyme inhibitor: acibenzolar acid [68]. Acibenzolar acid has only weak inhibitory effects on HCT activity, and a significant inhibitory effect could only be found with 300 μM acibenzolar acid. The use of acibenzolar acid had little influence on the symbiotic process but deepened the degree of mycelial infection. Under the 300 μM treatment, the depth of the Hartig net increased significantly, which may be because acibenzolar acid enlarges the intercellular spaces among cortical cells, which facilitates the infection of mycelium.

## 5. Conclusions

**Figure 9:**
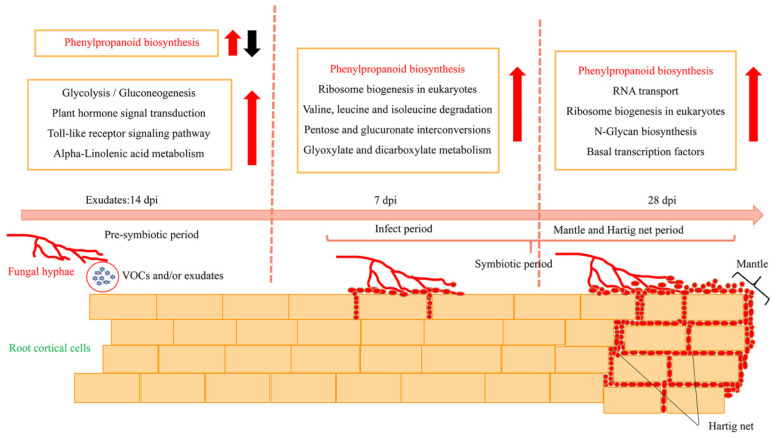
Schematic diagram showing morphological and transcriptional changes in a *P. massoniana* root during the formation of an ECM symbiosis with *S. bovinus*. Red arrows indicate upregulation; black arrow indicates downregulation. The phenylpropanoid biosynthesis pathway is common to all three ECM stages.

We investigated the characteristics of morphological and transcriptional changes during the establishment of the ECM symbiosis between *P. massoniana* and *S. bovinus*. ECM formation between *P. massoniana* roots and *S. bovinus* can be divided into two stages: the pre-symbiotic stage and the symbiotic stage (Figure 9). During the pre-symbiotic stage, VOCs and/or exudates released by *S. bovinus* induced host root growth. At 7 dpi, the mycelia invaded the intercellular space of the root cortex, and at 28 dpi, the functional ECM established with the mantle and Hartig net fully developed. Meanwhile, the biosynthesis of secondary metabolites, signal transduction, genetic information processing, and carbohydrate and lipid metabolism in *P. massoniana* roots changed in response to colonization by *S. bovinus*. The phenylpropanoid biosynthesis pathway was common to all three stages during the development of this ECM symbiosis, and the activity of a key enzyme-HCT-was related to the formation of the Hartig net. These findings highlight the need for a comprehensive investigation of the roles of the phenylpropanoid biosynthesis pathway in ECM formation, and perhaps also in ECM functions.

## Data Availability

The original data presented in this study are included in the article/Supplementary Materials. Further inquiries can be directed to the corresponding author.

## Supplementary Materials

The following supporting information can be downloaded at: https://www.mdpi.com/article/10.3390/jof8111162/s1. Figure S1: Effect of acibenzolar acid on the HCT enzyme activity of *P. massoniana*; Table S1: Quality analysis of transcriptome data; Table S2: HCT-and C3H-related genes at three stages of symbiosis.
