# Supplementary material for: Morphological and Transcriptional Characteristics of the Symbiotic Interaction between Pinus massoniana and Suillus bovinus
Source: J Fungi (Basel). 2022 Nov 3;8(11):1162. doi: 10.3390/jof8111162 (PMC9699607; doi:10.3390/jof8111162)
Supplement: Supplementary file 1 [file jof-08-01162-s001.zip › jof-1998856-supplementary.pdf]

Table S1. Quality analysis of transcriptome data

Sample	Raw reads	Clean reads	Clean bases (G)	Error (%)	Q20 (%)	Q30 (%)	GC (%)
Exud-1	34,669,458	33,293,211	9.99	0.03	97.79	93.76	44.09
Exud-2	41,352,829	39,671,968	11.90	0.03	97.79	93.77	43.97
Exud-3	41,689,066	39,797,465	11.94	0.03	97.85	93.91	44.16
Exud-4	43,966,585	42,806,796	12.84	0.03	97.78	93.70	43.81
NExud-1	35,180,772	34,264,628	10.28	0.03	97.11	92.26	44.91
NExud-2	35,746,298	34,673,391	10.40	0.03	97.87	94.00	44.83
NExud-3	34,873,724	34,058,344	10.22	0.03	97.65	93.53	44.68
NExud-4	35,521,178	34,550,601	10.37	0.03	97.67	93.54	44.67
M7-1	72,895,336	71,786,846	10.77	0.03	97.79	93.44	45.44
M7-2	72,804,880	71,467,912	10.72	0.03	97.66	93.26	45.36
M7-3	68,459,818	67,120,252	10.07	0.03	97.70	93.38	45.90
M7-4	66,600,994	65,381,004	9.81	0.03	97.71	93.39	46.05
NM7-1	69,181,450	67,762,212	10.16	0.03	97.50	92.92	45.17
NM7-2	67,465,120	66,173,142	9.93	0.03	97.39	92.67	44.85
NM7-3	67,762,262	66,439,738	9.97	0.03	97.57	93.06	44.95
NM7-4	69,875,092	68,322,248	10.25	0.03	97.49	92.92	44.99
M28-1	42,746,373	41,704,516	12.51	0.03	97.74	93.77	46.50
M28-2	42,531,059	41,317,397	12.40	0.03	97.88	94.05	45.82
M28-3	39,833,901	38,702,771	11.61	0.03	97.78	93.82	46.32
M28-4	40,599,494	39,346,443	11.80	0.03	97.87	94.02	46.41
NM28-1	35,153,620	34,121,932	10.24	0.03	97.84	93.93	44.57
NM28-2	35,456,958	34,431,853	10.33	0.03	97.78	93.75	44.95
NM28-3	37,461,503	36,336,922	10.90	0.03	97.73	93.72	44.91
NM28-4	35,136,937	34,097,514	10.23	0.03	97.72	93.67	44.84

Table S2. HCT and C3H synthesis-related genes at three stages of symbiosis

Symbiosis stages	Gene name	Gene ID	Description	Fold change Log_2 (M/NM)
7 dpi	HCT	*Cluster-20437.26389*	Shikimate *O*-hydroxycinnamoyltransferase	1.017
	C3H	*Cluster-20437.16414*	Coumaroyl quinate (coumaroyl shikimate) 3'-monooxygenase	12.063
		Cluster-20437.32796		7.584
		Cluster-20437.36203		9.759
		Cluster-20437.11543		11.902
		Cluster-20437.24065		11.761
		Cluster-20437.20940		9.629
		Cluster-20437.8480		10.069
		Cluster-20437.45220		10.150
		Cluster-20437.15558		11.576
28 dpi	HCT	*Cluster-21925.73278*	Shikimate *O*-hydroxycinnamoyltransferase	1.029
		Cluster-21925.72546		2.434
		Cluster-21925.23770		2.257
	C3H	*Cluster-21925.16352*	Coumaroyl quinate (coumaroyl shikimate) 3'-monooxygenase	12.503
		Cluster-21925.17949		12.931
		Cluster-21925.19083		10.973
		Cluster-21925.14804		8.039
		Cluster-21925.16762		10.821
		Cluster-21925.20271		10.572
		Cluster-21925.91767		12.538
		Cluster-21925.92800		11.860
		Cluster-21925.20289		12.628
		Cluster-21925.18995		8.791
		Cluster-21925.95793		9.264
		Cluster-21925.16527		11.449

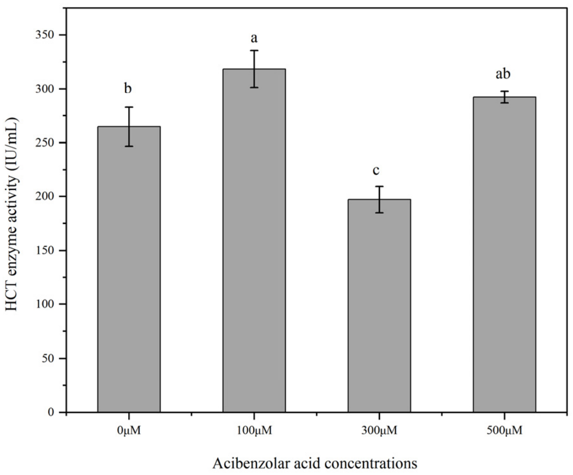

Figure S1. Effect of acibenzolar acid on the HCT enzyme activity of *P. massoniana*. Bars represent mean values ± the SE, $n = 4$; different letters above bars indicate significant differences between treatments at $p < 0.05$.
